# 2-Chloro-7-methyl­quinoline-3-carbaldehyde

**DOI:** 10.1107/S1600536809040823

**Published:** 2009-10-13

**Authors:** R. Subashini, F. Nawaz Khan, Rajesh Kumar, Venkatesha R. Hathwar, Seik Weng Ng

**Affiliations:** a Chemistry Division, School of Science and Humanities, VIT University, Vellore 632 014, Tamil Nadu, India; b Solid State and Structural Chemistry Unit, Indian Institute of Science, Bangalore 560 012, Karnataka, India; c Department of Chemistry, University of Malaya, 50603 Kuala Lumpur, Malaysia

## Abstract

The quinoline fused-ring system of the title compound, C_11_H_8_ClNO, is planar (r.m.s. deviation = 0.007 Å); the formyl group is bent slightly out of the plane [C—C—C—O torsion angles = −9.6 (5) and 170.4 (3)°].

## Related literature

For a review of the synthesis of quinolines by the Vilsmeier–Haack reaction, see: Meth-Cohn (1993 ▶).
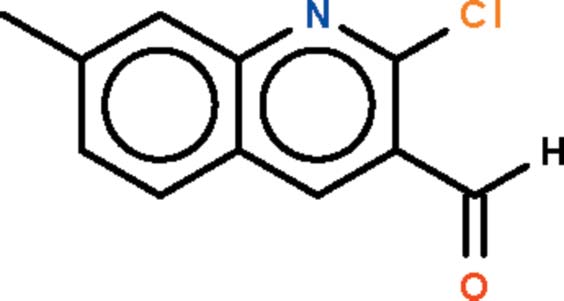


## Experimental

### 

#### Crystal data

C_11_H_8_ClNO
*M* *_r_* = 205.63
Monoclinic, 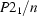

*a* = 15.458 (3) Å
*b* = 3.9382 (8) Å
*c* = 16.923 (3) Å
β = 112.854 (3)°
*V* = 949.3 (3) Å^3^
*Z* = 4
Mo *K*α radiation
μ = 0.36 mm^−1^
*T* = 290 K
0.24 × 0.18 × 0.06 mm

#### Data collection

Bruker SMART area-detector diffractometer
Absorption correction: multi-scan (*SADABS*; Sheldrick, 1996 ▶) *T*_min_ = 0.918, *T*_max_ = 0.979
6484 measured reflections
1796 independent reflections
1356 reflections with *I* > 2σ(*I*)
*R*_int_ = 0.042

#### Refinement

*R*[*F* ^2^ > 2σ(*F* ^2^)] = 0.078
*wR*(*F* ^2^) = 0.209
*S* = 1.13
1796 reflections
128 parameters
H-atom parameters constrained
Δρ_max_ = 0.78 e Å^−3^
Δρ_min_ = −0.49 e Å^−3^

### 

Data collection: *SMART* (Bruker, 2004 ▶); cell refinement: *SAINT* (Bruker, 2004 ▶); data reduction: *SAINT*; program(s) used to solve structure: *SHELXS97* (Sheldrick, 2008 ▶); program(s) used to refine structure: *SHELXL97* (Sheldrick, 2008 ▶); molecular graphics: *X-SEED* (Barbour, 2001 ▶); software used to prepare material for publication: *publCIF* (Westrip, 2009 ▶).

## Supplementary Material

Crystal structure: contains datablocks global, I. DOI: 10.1107/S1600536809040823/xu2629sup1.cif

Structure factors: contains datablocks I. DOI: 10.1107/S1600536809040823/xu2629Isup2.hkl

Additional supplementary materials:  crystallographic information; 3D view; checkCIF report

## Figures and Tables

**Fig. 1.**
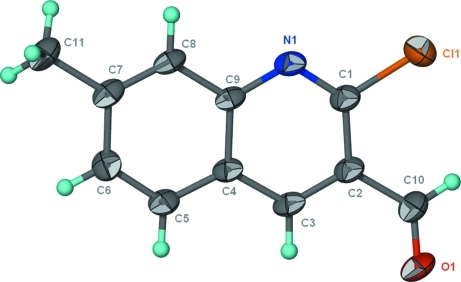
Thermal ellipsoid plot (Barbour, 2001) of C11H8ClNO at the 50% probability level; hydrogen atoms are drawn as spheres of arbitrary radius.

## supplementary crystallographic information

### Experimental

A Vilsmeier-Haack adduct prepared from phosphorus oxytrichloride (6.5 ml, 70 mmol) and *N*,*N*-dimethylformamide (2.3 ml, 30 mmol) at 273 K was added *N*-(3-tolyl)acetamide (1.49 g, 10 mmol). The mixture was heated at 353 K for 15 h. The mixture was poured onto ice; the white product was collected and dried. The compound was purified by recrystallization from a petroleum ether/ethyl acetate mixture.

### Refinement

Carbon-bound H-atoms were placed in calculated positions (C–H 0.93–0.96 Å) and were included in the refinement in the riding model approximation, with *U*(H) set to 1.2–1.5*U*(C).

### Crystal data

**Table:**

C_11_H_8_ClNO	*F*(000) = 424
*M**_r_* = 205.63	*D*_x_ = 1.439 Mg m^−^^3^
Monoclinic, *P*2_1_/*n*	Mo *K*α radiation, λ = 0.71073 Å
Hall symbol: -P 2yn	Cell parameters from 973 reflections
*a* = 15.458 (3) Å	θ = 1.3–24.9°
*b* = 3.9382 (8) Å	µ = 0.36 mm^−^^1^
*c* = 16.923 (3) Å	*T* = 290 K
β = 112.854 (3)°	Block, colorless
*V* = 949.3 (3) Å^3^	0.24 × 0.18 × 0.06 mm
*Z* = 4	

### Data collection

**Table:**

Bruker SMART area-detector diffractometer	1796 independent reflections
Radiation source: fine-focus sealed tube	1356 reflections with *I* > 2σ(*I*)
graphite	*R*_int_ = 0.042
φ and ω scans	θ_max_ = 25.7°, θ_min_ = 2.3°
Absorption correction: multi-scan (*SADABS*; Sheldrick, 1996)	*h* = −18→18
*T*_min_ = 0.918, *T*_max_ = 0.979	*k* = −4→4
6484 measured reflections	*l* = −20→20

### Refinement

**Table:**

Refinement on *F*^2^	Primary atom site location: structure-invariant direct methods
Least-squares matrix: full	Secondary atom site location: difference Fourier map
*R*[*F*^2^ > 2σ(*F*^2^)] = 0.078	Hydrogen site location: inferred from neighbouring sites
*wR*(*F*^2^) = 0.209	H-atom parameters constrained
*S* = 1.13	*w* = 1/[σ^2^(*F*_o_^2^) + (0.1371*P*)^2^] where *P* = (*F*_o_^2^ + 2*F*_c_^2^)/3
1796 reflections	(Δ/σ)_max_ = 0.001
128 parameters	Δρ_max_ = 0.78 e Å^−^^3^
0 restraints	Δρ_min_ = −0.49 e Å^−^^3^

### Fractional atomic coordinates and isotropic or equivalent isotropic displacement parameters (Å^2^)

**Table:**

	*x*	*y*	*z*	*U*_iso_*/*U*_eq_	
Cl1	0.37647 (6)	0.6903 (3)	0.18658 (6)	0.0603 (4)	
O1	0.36833 (17)	0.1214 (8)	0.39875 (18)	0.0705 (9)	
N1	0.55664 (19)	0.6719 (7)	0.27097 (16)	0.0402 (7)	
C1	0.4781 (2)	0.5835 (8)	0.27548 (19)	0.0393 (7)	
C2	0.4683 (2)	0.4068 (8)	0.34482 (19)	0.0383 (7)	
C3	0.5497 (2)	0.3312 (8)	0.4129 (2)	0.0387 (7)	
H3	0.5468	0.2182	0.4601	0.046*	
C4	0.6373 (2)	0.4210 (7)	0.41281 (18)	0.0347 (7)	
C5	0.7243 (2)	0.3490 (8)	0.48060 (19)	0.0407 (8)	
H5	0.7253	0.2376	0.5294	0.049*	
C6	0.8064 (2)	0.4414 (8)	0.47489 (19)	0.0424 (8)	
H6	0.8628	0.3923	0.5201	0.051*	
C7	0.8080 (2)	0.6125 (7)	0.4009 (2)	0.0394 (8)	
C8	0.7248 (2)	0.6851 (8)	0.3354 (2)	0.0391 (7)	
H8	0.7252	0.7978	0.2872	0.047*	
C9	0.6379 (2)	0.5927 (7)	0.33897 (18)	0.0341 (7)	
C10	0.3769 (2)	0.3059 (9)	0.3458 (2)	0.0503 (9)	
H10	0.3228	0.3900	0.3029	0.060*	
C11	0.9001 (3)	0.7114 (9)	0.3968 (2)	0.0523 (9)	
H11A	0.8906	0.9001	0.3584	0.078*	
H11B	0.9248	0.5226	0.3764	0.078*	
H11C	0.9436	0.7746	0.4530	0.078*	

### Atomic displacement parameters (Å^2^)

**Table:**

	*U*^11^	*U*^22^	*U*^33^	*U*^12^	*U*^13^	*U*^23^
Cl1	0.0499 (6)	0.0826 (8)	0.0457 (6)	0.0050 (4)	0.0157 (4)	0.0154 (4)
O1	0.0512 (17)	0.103 (2)	0.0731 (18)	−0.0030 (14)	0.0416 (15)	0.0286 (15)
N1	0.0478 (16)	0.0474 (15)	0.0341 (14)	0.0010 (12)	0.0252 (13)	0.0033 (10)
C1	0.0427 (18)	0.0452 (17)	0.0380 (16)	0.0017 (13)	0.0245 (14)	0.0012 (13)
C2	0.0407 (18)	0.0452 (17)	0.0395 (17)	0.0026 (12)	0.0272 (14)	0.0014 (12)
C3	0.0476 (19)	0.0439 (17)	0.0383 (16)	0.0018 (13)	0.0316 (15)	0.0017 (12)
C4	0.0424 (17)	0.0396 (15)	0.0323 (15)	0.0025 (12)	0.0257 (13)	−0.0005 (11)
C5	0.0439 (18)	0.0549 (19)	0.0338 (16)	0.0035 (14)	0.0267 (14)	0.0011 (13)
C6	0.0390 (17)	0.0560 (19)	0.0386 (17)	0.0053 (14)	0.0221 (14)	−0.0053 (14)
C7	0.0467 (19)	0.0403 (16)	0.0449 (18)	−0.0047 (13)	0.0328 (16)	−0.0095 (12)
C8	0.0477 (19)	0.0440 (17)	0.0400 (17)	−0.0032 (13)	0.0327 (15)	−0.0016 (12)
C9	0.0423 (17)	0.0391 (15)	0.0322 (15)	−0.0007 (12)	0.0268 (13)	−0.0019 (11)
C10	0.0388 (19)	0.065 (2)	0.055 (2)	0.0019 (15)	0.0272 (17)	0.0066 (17)
C11	0.0469 (19)	0.060 (2)	0.063 (2)	−0.0075 (15)	0.0351 (17)	−0.0042 (16)

### Geometric parameters (Å, °)

**Table:**

Cl1—C1	1.753 (3)	C5—H5	0.9300
O1—C10	1.200 (4)	C6—C7	1.430 (4)
N1—C1	1.293 (4)	C6—H6	0.9300
N1—C9	1.370 (4)	C7—C8	1.363 (4)
C1—C2	1.422 (4)	C7—C11	1.502 (5)
C2—C3	1.369 (4)	C8—C9	1.416 (4)
C2—C10	1.473 (4)	C8—H8	0.9300
C3—C4	1.400 (4)	C10—H10	0.9300
C3—H3	0.9300	C11—H11A	0.9600
C4—C5	1.417 (4)	C11—H11B	0.9600
C4—C9	1.424 (4)	C11—H11C	0.9600
C5—C6	1.359 (4)		
			
C1—N1—C9	117.7 (3)	C8—C7—C6	118.6 (3)
N1—C1—C2	125.7 (3)	C8—C7—C11	121.3 (3)
N1—C1—Cl1	115.7 (2)	C6—C7—C11	120.1 (3)
C2—C1—Cl1	118.5 (2)	C7—C8—C9	121.5 (3)
C3—C2—C1	116.3 (3)	C7—C8—H8	119.3
C3—C2—C10	120.2 (3)	C9—C8—H8	119.3
C1—C2—C10	123.5 (3)	N1—C9—C8	118.8 (3)
C2—C3—C4	121.2 (3)	N1—C9—C4	121.9 (3)
C2—C3—H3	119.4	C8—C9—C4	119.3 (3)
C4—C3—H3	119.4	O1—C10—C2	123.8 (3)
C3—C4—C5	124.3 (3)	O1—C10—H10	118.1
C3—C4—C9	117.2 (3)	C2—C10—H10	118.1
C5—C4—C9	118.5 (3)	C7—C11—H11A	109.5
C6—C5—C4	120.5 (3)	C7—C11—H11B	109.5
C6—C5—H5	119.7	H11A—C11—H11B	109.5
C4—C5—H5	119.7	C7—C11—H11C	109.5
C5—C6—C7	121.5 (3)	H11A—C11—H11C	109.5
C5—C6—H6	119.3	H11B—C11—H11C	109.5
C7—C6—H6	119.3		
			
C9—N1—C1—C2	−0.7 (5)	C5—C6—C7—C11	179.9 (3)
C9—N1—C1—Cl1	−179.8 (2)	C6—C7—C8—C9	−0.5 (4)
N1—C1—C2—C3	1.3 (5)	C11—C7—C8—C9	−180.0 (3)
Cl1—C1—C2—C3	−179.6 (2)	C1—N1—C9—C8	179.6 (3)
N1—C1—C2—C10	−178.7 (3)	C1—N1—C9—C4	−0.4 (4)
Cl1—C1—C2—C10	0.5 (4)	C7—C8—C9—N1	−179.8 (3)
C1—C2—C3—C4	−0.8 (4)	C7—C8—C9—C4	0.2 (4)
C10—C2—C3—C4	179.2 (3)	C3—C4—C9—N1	0.8 (4)
C2—C3—C4—C5	−179.5 (3)	C5—C4—C9—N1	−179.8 (3)
C2—C3—C4—C9	−0.2 (4)	C3—C4—C9—C8	−179.2 (3)
C3—C4—C5—C6	179.1 (3)	C5—C4—C9—C8	0.1 (4)
C9—C4—C5—C6	−0.2 (4)	C3—C2—C10—O1	−9.6 (5)
C4—C5—C6—C7	−0.1 (5)	C1—C2—C10—O1	170.4 (3)
C5—C6—C7—C8	0.5 (5)		
